# A self-assembling split Nano luciferase-based assay for investigating *Pseudomonas syringae* effector secretion

**DOI:** 10.1007/s44154-024-00152-2

**Published:** 2024-02-16

**Authors:** Pei Miao, Jian-Min Zhou, Wei Wang

**Affiliations:** 1 State Key Laboratory of Plant Genomics, Institute of Genetics and Developmental Biology, Chinese Academy of Sciences, Beijing, 100101 China; 2 College of Advanced Agricultural Sciences, University of Chinese Academy of Sciences, Beijing, 100049 China; 3 CAS Center for Excellence in Biotic Interactions, University of Chinese Academy of Sciences, Beijing, 100049 China; 4 Yazhouwan National Laboratory, Sanya, 572024 China

**Keywords:** *Pseudomonas syringae*, T3SS effectors, Secretion, Nano luciferase

## Abstract

Many Gram-negative pathogens employ the type III secretion system (T3SS) to deliver effector proteins into host cells, thereby modulating host cellular processes and suppressing host immunity to facilitate pathogenesis and colonization. In this study, we developed a straightforward, rapid, and quantitative method for detecting T3SS-mediated translocation of *Pseudomonas syringae* effectors using a self-assembling split Nano luciferase (Nluc)-based reporter system. It was demonstrated that this system can detect effector secretion in vitro with an exceptionally high signal-to-noise ratio and sensitivity, attributed to the strong affinity between the split domains of Nluc and the intense luminescence generated by functional Nluc. During natural infections, effectors fused to a small C-terminal fragment of Nluc were successfully translocated into plant cells and retained their virulence functions. Furthermore, upon infection of plants expressing the N-terminal fragment of Nluc with these *P. syringae* strains, functional Nluc proteins were spontaneously assembled and produced bright luminescence, demonstrating that this system enables the straightforward and rapid assessment of *P. syringae* T3SS-mediated effector translocation during natural infections. In conclusion, the self-assembling split Nluc-based reporting system developed in this study is suitable for efficient in vitro and *in planta* detection of effectors secreted via T3SS.

**Supplementary Information:**

The online version contains supplementary material available at 10.1007/s44154-024-00152-2.

## Main text

Many Gram-negative plant pathogenic bacteria employ the type III secretion system (T3SS) to translocate virulence effectors into host cells (Xin et al. 2018). These effectors manipulate various host cellular processes to facilitate pathogenesis and host colonization (Wang et al. 2022). As the core of bacterial virulence, the transcription and secretion of effectors are regulated by plants and environmental conditions (Anderson 2023; Kvitko and Collmer 2023). Therefore, monitoring the secretion of effectors is crucial for understanding the mechanisms underlying plant–bacteria interactions. The secretion of *Pseudomonas syringae* effectors in vitro is typically detected by immunoblot analysis using specific antibodies (Crabill et al. 2010; Jin and He 2001). However, this method has limitation such as low sensitivity, qualitative, and unsuitability for high-throughput studies. A split-green fluorescent protein (GFP) complementation strategy and a calmodulin-dependent adenylate cyclase (Cya) assay were designed to examine the translocation of *P. syringae* effectors during natural infections (Casper-Lindley et al. 2002; Henry et al. 2017; Park et al. 2017; Schechter et al. 2004). However, the low sensitivity and slow kinetics of the split-GFP complementation strategy limit its application for analyzing translocation kinetics. While the Cya-based system allows for the quantitative detection of effector secretion, it remians labor-intensive and costly.

**Fig. 1:**
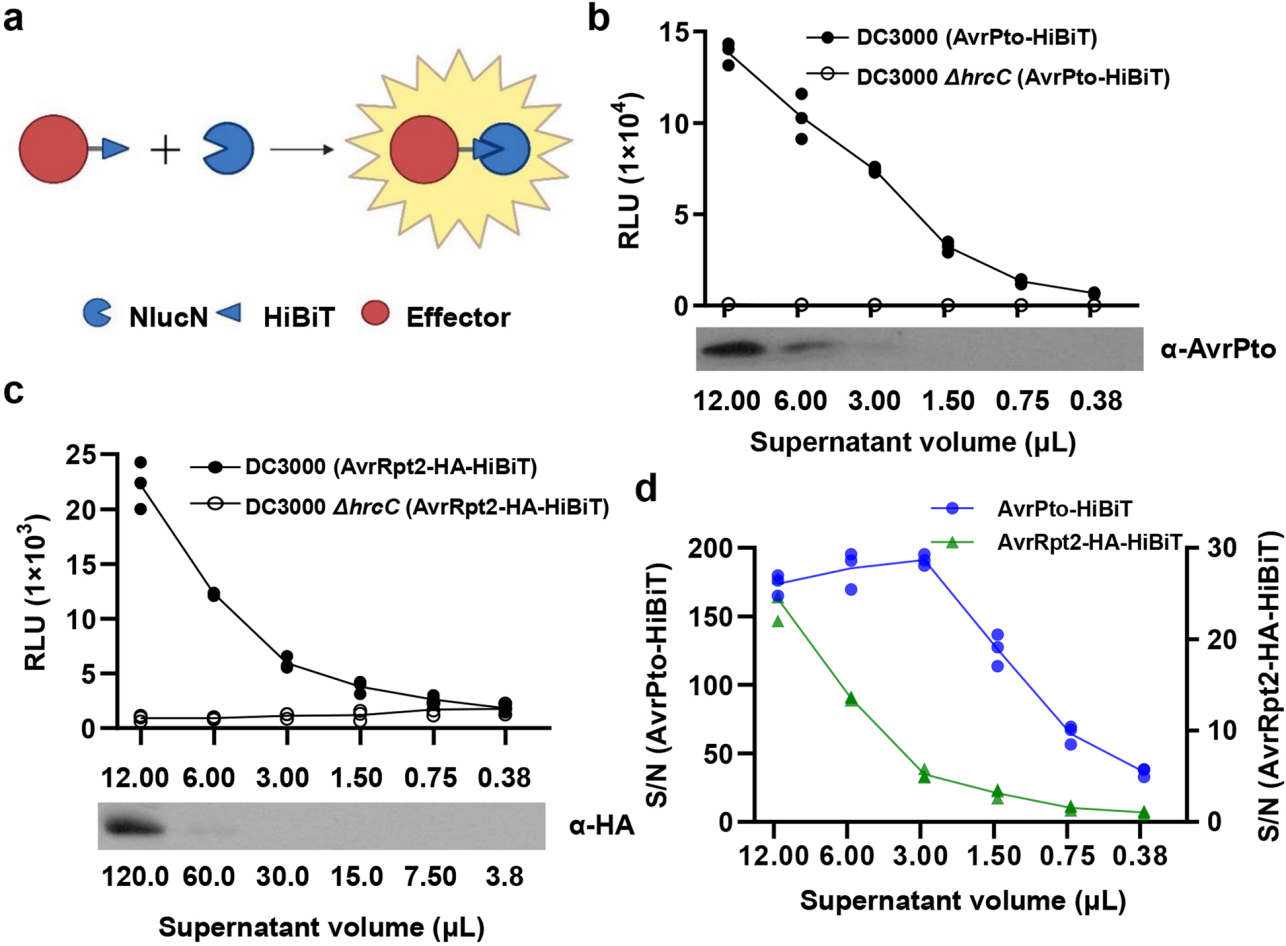
Examination of bacterial effector secretion in vitro using a self-assembling split Nluc-based reporting system*.* **a** Diagram of the Nluc-based split luciferase complementation assay. HiBiT, which displays high affinity to NlucN, is fused to the C terminus of a bacterial effector of interest via a flexible linker. The effector-HiBiT fusions spontaneously associate with NlucN to produce a functional Nluc. **b**–**d** Secretion of AvrPto-HiBiT and AvrRpt2-HA-HiBiT in vitro. AvrPto-HiBiT (**b**) and AvrRpt2-HA-HiBiT (**c**) were detected in serially diluted culture supernatants of *Pst* DC3000 but not the *Pst* DC3000 *ΔhrcC* mutant, based on reconstitution of a functional Nluc. Expression and secretion of the indicated effectors was induced in vitro, and the specified volumes of culture supernatant were collected, incubated with coelenterazine *h* and NlucN-His recombinant protein, and measured for luminescence. Total protein from the indicated samples was analyzed by immunoblot using anti-AvrPto and anti-HA antibodies to detect the accumulation of AvrPto-HiBiT (**b**) and AvrRpt2-HA-HiBiT (**c**), respectively. **d** Signal-to-noise ratio (S/N) for each sample in **b** and **c**. The S/N of each sample was calculated as the ratio between the luminescence for *Pst* DC3000 and the luminescence for *Pst* DC3000 *ΔhrcC*. In (**b** to **d**) three replicates were performed per sample, with the data line linking the mean values

Nano luciferase (Nluc), an engineered enzyme (approximately 19 kDa) derived from the small subunit of the deep-sea shrimp *Oplophorus gracilirostris* luciferase, exhibits superior physical stability and brighter luminescence compared to luciferases from firefly (*Photinus pyralis*) or the sea pansy (*Renilla reniformis*) (Hall et al. 2012; England et al. 2016). Nluc can be split into a larger N-terminal fragment (NlucN, 159 amino acids) and a smaller C-terminal fragment (NlucC, 11 amino acids). HiBiT, an NlucC variant comprising 11 amino acids, spontaneously binds to NlucN with high affinity, leading to the assembly of active Nluc (Dixon et al. 2016) (Fig. 1a). This self-assembling split Nano luciferase (Nluc) reporting system has been successfully used to detect the secretion of *Salmonella* effectors in vitro or into host cells (Westerhausen et al. 2020).

To evaluate the potential application of this system in quantifying effector secretion by the plant pathogenic bacteria *P. syringae*, we fused the HiBiT peptide to the C terminus of AvrPto or HA-tagged AvrRpt2 via a flexible linker (GGSGG). The effector-HiBiT fusions were cloned into the pUCP20 plasmid under the control of their respective native promoters. The resulting constructs were then transformed into the *P. syringae* wild-type strain *Pst* DC3000 or a T3SS-deficient mutant strain *Pst* DC3000 *ΔhrcC*. After inducing effector expression and secretion in vitro, we collected aliquots of cultures and filtered them to obtain cell-free supernatants. The secretion of AvrPto or AvrRpt2 was assessed using the self-assembling split Nluc reporting system and immunoblot analysis, respectively. While luminescence was detected in the filtered supernatants of *Pst* DC3000 (AvrRpt2-HA-HiBiT) and *Pst* DC3000 (AvrPto-HiBiT) cultures when incubated with NlucN-His recombinant protein and coelenterazine *h*, the substrate of Nluc, it was undetectable when using filtered supernatants from comparable cultures of the *Pst* DC3000 *ΔhrcC* mutant (Fig. 1b and 1c), indicating a T3SS-mediated secretion of AvrPto and AvrRpt2. Moreover, the signal-to-noise ratio (luminescence of *Pst* DC3000/ luminescence of *Pst* DC3000 *ΔhrcC*; S/N) for AvrRpt2 and AvrPto remained higher than 5, even with as little as 3 μL of the filtered culture supernatant (Fig. 1d).

We compared the sensitivity and time efficiency of immunoblotting to the self-assembling split Nluc-based assay. AvrPto-HiBiT and AvrRpt2-HA-HiBiT were successfully detected in filtered culture supernatants using an immunoblot-based assay in culture aliquots of 6 μL and 120 μL, respectively, but not in smaller volumes. Importantly, the entire procedure took at least 6 h (Fig. 1b and c). In contrast, the secretion of AvrPto-HiBiT and AvrRpt20-HA-HiBiT (S/N > 5) could be assessed within 30 min via the self-assembling split Nluc-based reporter assay, and the minimum required volume of filtered culture supernatant was only 0.38 μL or 3 μL, respectively (Fig. 1d). Taken together, these results indicate that *P. syringae* effector-HiBiT fusions were successfully secreted in vitro and could be quickly and efficiently detected in culture supernatants using the self-assembling split Nluc-based reporting system.

**Fig. 2:**
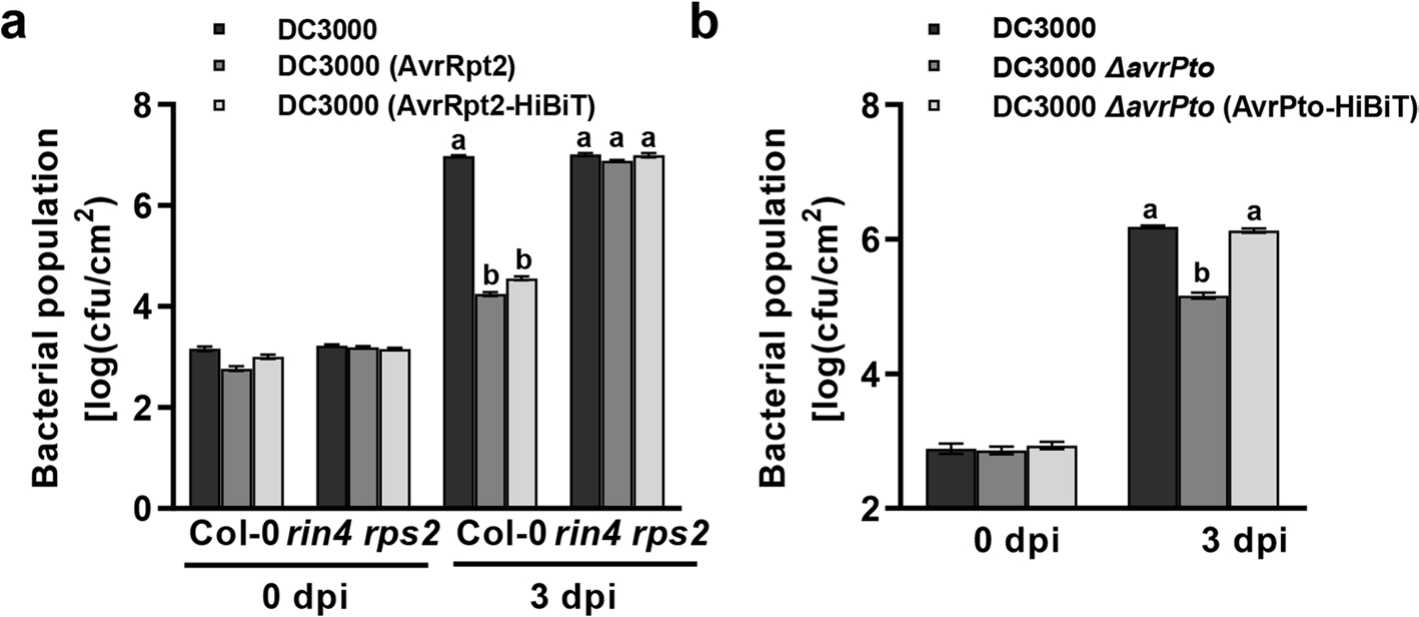
Fusion of effectors to HiBiT does not affect their biological activity. **a** AvrRpt2 and AvrRpt2-HiBiT are similarly recognized by RPS2. *Pst* DC3000 and *Pst* DC3000 carrying *avrRpt2* or *avrRpt2-HiBiT* were infiltrated into the leaves of indicated plants and bacterial growth was assessed 3 days later. **b** The attenuated virulence of *Pst* DC3000 caused by the *avrPto* mutation is rescued by AvrPto*-*HiBiT. Col-0 leaves were infiltrated with the indicated bacterial suspensions, and bacterial titers were determined 3 days later. Data in (**a** and **b**) are means ± SE, different letters indicate significant differences at *P* ≤ 0.05 (n ≥ 6, one-way ANOVA, Tukey post-test)

To assess the suitability of the Nluc-based assay for measuring *P. syringae* effector secretion *in planta,* we investigated whether the virulence function of the effectors was affected by their fusion to HiBiT. The *P. syringae* effector AvrRpt2 is recognized by the nucleotide-binding leucine-rich repeat (NLR) receptor RESISTANT TO P. SYRINGAE 2 (RPS2) (Kunkel et al. 1993; Yu et al. 1993). When inoculated into *Arabidopsis thaliana* wild-type Col-0, *Pst* DC3000 carrying *avrRpt2* and *Pst* DC3000 carrying *avrRpt2-HiBiT* showed decreased bacterial growth compared to *Pst* DC3000. However, we did not observe this effector-mediated decrease in bacterial growth after inoculation into the Arabidopsis* rin4 rps2* double mutant (Fig. 2a). AvrPto is one of the most virulence effectors of *Pst* DC3000. Knocking out *avrPto* in *Pst* DC3000 results in a significant decrease in bacterial growth during infection (Xiang et al. 2008), but this phenotype was rescued by the expression of AvrPto-HiBiT (Fig. 2b). These results indicate that effector-HiBit fusion proteins are functional *in planta* and can be successfully delivered into host cells by the T3SS of *Pst* DC3000.

**Fig. 3:**
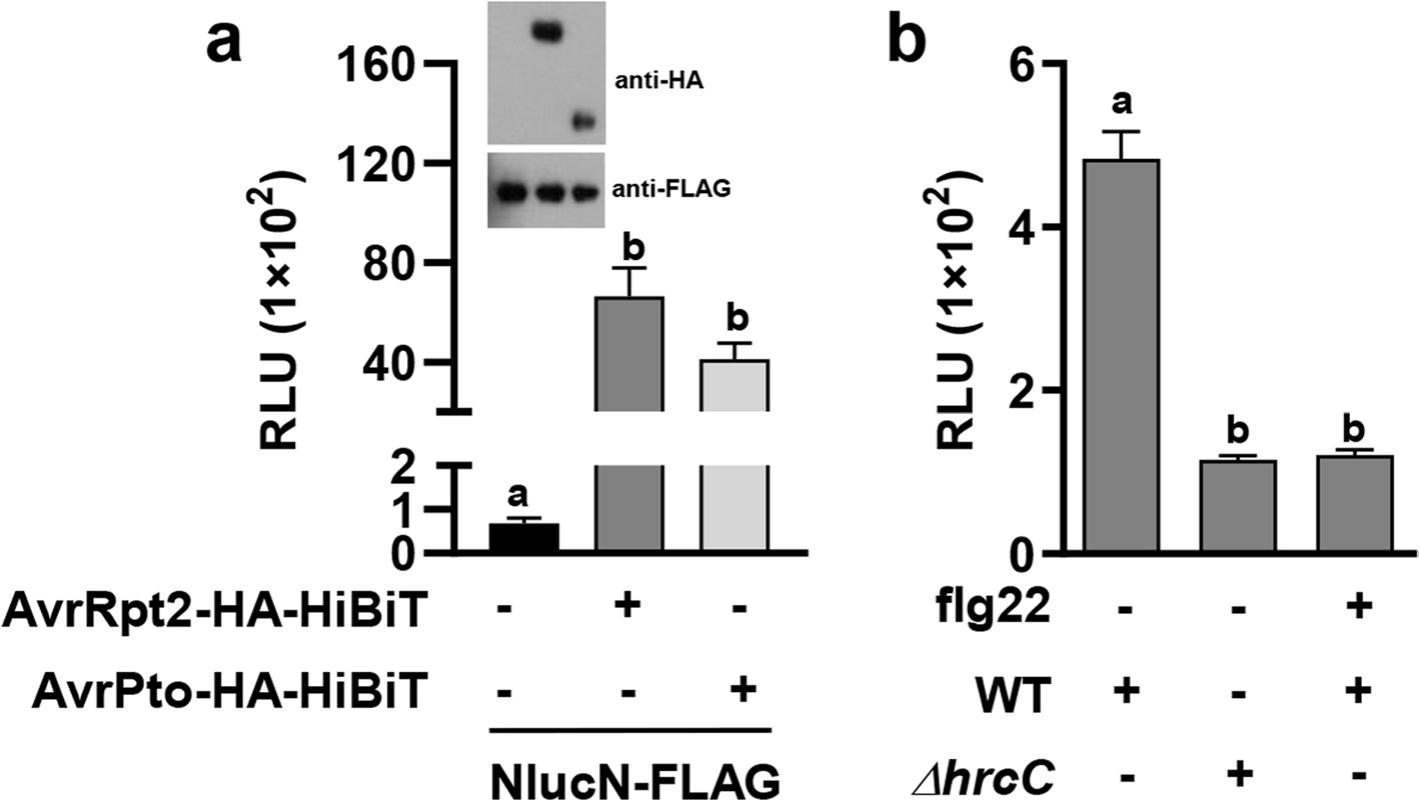
Detection of bacterial effector translocation *in planta* using the self-assembling split Nluc-based reporting system. **a** Complementation of split Nluc in *N. benthamiana*. Co-expression of AvrRpt2-HA-HiBiT or AvrPto-HA-HiBiT with NlucN results in the reconstitution of a functional Nluc. The indicated constructs were infiltrated in the leaves of *N. benthamiana* plants; NLuc activity and the expression of indicated protein were determined. **b** Secretion of AvrPto-HiBiT in Arabidopsis. The leaves from transgenic Arabidopsis lines expressing *NlucN* were infiltrated with H_2_O or 1 μM flg22. After 24 h, the indicated bacteria were inoculated onto the same leaves, leaf discs were harvested 9 h after the secondary infiltration for luminescence measurements

We further examined whether the co-expression of HiBiT and NlucN *in planta* can reconstitute a functional Nluc luciferase. To this end, HiBiT-effectors fusions were co-expressed with NlucN in *Nicotiana benthamiana* and the intensity of luminescence was determined. Luciferase activity was significantly higher in samples co-expressing NlucN with either AvrRpt2-HA-HiBiT or AvrPto-HA-HiBiT, compared to those expressing NlucN alone (Fig. 3a). These results indicate that the co-expression of effector-HiBiT and NlucN leads to the spontaneous self-assembly of a functional luciferase *in planta*.

We also evaluated the applicability of the self-assembling split Nluc-based reporting system for tracking effector translocation during natural infections. Leaves of Arabidopsis transgenic plants constitutively expressing *NlucN* were inoculated with either *Pst* DC3000 (AvrPto-HiBiT) or *Pst* DC3000 *ΔhrcC* (AvrPto-HiBiT). Luciferase activity in the leaves inoculated with *Pst* DC3000 was signifcantly higher than in those inoculated with the *ΔhrcC* mutant, indicating that T3SS-mediated AvrPto secretion was efficiently detected via the Nluc-based system (Fig. 3b). Furthermore, luminescence was significantly reduced in leaves pretreated with flg22 compared to leaves inoculated with *Pst* DC3000 (AvrPto-HiBiT) alone (Fig. 3b). These results are consistent with the repression of effector secretion by *P. syringae* due to pathogen-associated molecular pattern (PAMP)-triggered immunity (PTI) (Crabill et al. 2010; Oh et al. 2010). Collectively, our results demonstrate that the self-assembling Nluc reporter-based system is a valuable assay for examining the secretion of *P. syringae* effectors during natural infections.

Our study developed a self-assembling split Nluc-based assay to detect the secretion of bacterial effectors in vitro and their delivery into plant cells *in planta*. The high affinity between the two split domains of Nluc (*K*_*D*_ = 900 nM) and the intense luminescence generated by reconstituted Nluc makes this assay possess a remarkably high signal-to-noise ratio and sensitivity, even when examining effector secretion in microliter sample volumes in the inducing medium. Effector-HiBiT fusions were successfully translocated into host cells during natural infections and retained their virulence function, due to the small size of the HiBiT peptide. The robust reaction between Nluc and its substrate coelenterazine *h* enable this assay to be conducted under various conditions, such as in the culture medium or in plant leaves. This simple, highly sensitive, and cost-effective method could be employed for high-throughput, in vitro screening of synthetic compounds or natural metabolites as potential inhibitors of T3SS. It also can be used to investigate the underlying mechanisms through which hosts regulate the secretion of bacterial effectors during natural infections.

## Materials and methods

### Plant materials and growth conditions

Transgenic lines expressing *NlucN* were generated in *Arabidopsis* *thaliana* wild type Col-0 background. *A. thaliana* and *Nicotiana benthamiana* plants were grown in nutrient soil at 23 °C with a 10/14- hour light/dark photoperiod.

### Bacterial strains

The bacterial strains used in this study include *P. syringae* pv. *tomato* DC3000 (*Pst* DC3000) (Zheng et al. 2022), *Pst* DC3000 *ΔavrPto* mutant strain (Xiang et al. 2008), *Pst* DC3000 carrying *avrRpt2* (Kunkel et al. 1993), *avrPto-HiBiT*, *or avrRpt2-HA-HiBiT*, *Pst* DC3000 *ΔhrcC* carrying *avrPto*-*HiBiT or avrRpt2-HA-HiBiT* (this study).

### Plasmid construction and transformation

A synthetic fragment of linker-HiBiT was inserted in pUCP20 using ClonExpress MultiS One Step Cloning Kit (Vazyme), the resulting new construct was named pUCP20-HiBiT. Subsequently, the promotor and coding sequences of *avrRpt2-HA* and *avrPto* were amplified and cloned into pUCP20 to generate pUCP20*-avrRpt2-HA*-HiBiT and pUCP20-*avrPto*-HiBiT, respectively. Then, the indicted construct was transformed into *Pst* DC3000 or *Pst* DC3000 *ΔhrcC* using electroporation.

To express the NlucN-His fusion protein in *E. coli,* a synthetic fragment of *NlucN* was inserted into pET28a, which was then transformed into BL21.

For the expression of AvrRpt2-HA-HiBiT, AvrPto-HA-HiBiT and NlucN-FLAG in *N. benthamiana*, the respective fragment was amplified from a template, and then inserted into pCAMBIA-1300 using ClonExpress MultiS One Step Cloning Kit (Vazyme). The resulting constructs were transformed into Agrobacterium *(Agrobacterium tumefaciens)* by electroporation. Additionally, pCAMBIA-1300-NlucN construct was introduced into the Col-0 background, and transgenic line expressing NlucN was determined in T1 plants by PCR analysis, then the homozygotes lines were identified in T2 generation and used in further experiments.

All primers used are listed in Table S1.

### Nluc-based complementation assay in *N. benthamiana*

For validation of self-assembling split Nluc in *N. benthamiana*, *A. tumefaciens* strain carrying *NlucN-FLAG,* along with *avrRpt2-HA-HiBiT* or *avrPto-HA-HiBiT*, were infiltrated into the leaves. Thirty hours after infiltration, leaves discs were collected and incubated with 10 µM coelenterazine *h* (MedChemExpress) in a 96-well plate for 10 min, then the luminescence was monitored using EnSpire Multimode Plate Reader (PerkinElmer). The total proteins of each sample were extracted and subjected to examine the expression of indicated proteins.

### Bacterial growth assay

Bacteria were cultured overnight at 28 °C in King’s B medium containing appropriate antibiotics. Bacteria were harvested, washed twice by sterile water and diluted to a final density OD600 = 0.001. Fully expanded and healthy leaves of 4-week-old plants were infiltrated with indicated bacterial suspensions. Bacterial populations in leaves were determined 3 days after inoculation.

### Analysis of effector secretion in vitro

To determine effector secretion by Nluc-based complementation in vitro, effectors expression and secretion was induced as described previously (Wang et al. 2020). Bacterial suspensions were centrifuged at 13,000 rpm for 10 min, and the supernatant subsequently filtered through a 0.22 μm pore to remove bacteria. The indicated volumes of the supernatants were then incubated with 10 μM coelenterazine *h* (MedChemExpress) and 10 μg purified NlucN-His protein in a 96-well plate for 10 min. The luminescence was monitored using EnSpire Multimode Plate Reader (PerkinElmer). The total protein from supernatants was analyzed by immunoblotting using anti-AvrPto and anti-HA antibodies to determine the secretion of AvrPto and AvrRpt2, respectively.

### Analysis of effector translocation *in planta*

To determine effector secretion by the Nluc-based complementation *in planta*, leaves of transgenic *A. thaliana* plants expressing *NlucN* were infiltrated with H_2_O or 1 μM flg22. Twenty-four hours after first infiltration, indicated bacteria (OD600 = 0.1) was infiltrated to the same leaves. Leaf discs were harvested 9 h later, and incubated with 10 μM Coelenterazine *h* (MedChemExpress) in a 96-well plate for 10 min, then the luminescence was monitored using EnSpire Multimode Plate Reader (PerkinElmer).

### Purification of recombinant proteins

For purification of NlucN-His, *E. coli* BL21 bacteria carrying *NlucN-His* were grown in LB medium at 28 °C until the OD600 reached 0.4. The expression of NlucN-His was then induced with 0.5 mM isopropyl-β-Dthiogalactopyranoside (IPTG; Sigma) at 16 °C for 16 h. After incubation, bacteria were collected and resuspended in lysis buffer (50 mM Tris–HCl pH 7.5, 300 mM sodium chloride, 10 mM imidazole), sonicated at 35% amplitude for 10 min (5 s on and 5 s off). Cell debris was removed by centrifugation at 16,000 rpm at 4 °C for 30 min, the supernatant was then subjected onto 1 mL of Ni–NTA agarose beads pre-equilibrated with lysis buffer. After being washed twice with wash buffer (50 mM Tris–HCl pH 7.5, 300 mM sodium chloride, 20 mM imidazole), NlucN-His recombinant protein was eluted with elution buffer (50 mM Tris–HCl pH 7.5, 300 mM sodium chloride, 250 mM imidazole). Finally, the eluted Nluc-His protein was further diluted with buffer (50 mM Tris–HCl pH 8.0, 100 mM sodium chloride, 10% glycerol) to remove imidazole and concentrated to about 1 mg/ml.

### Supplementary Information

**Additional file 1: Table S1.** Primers used in this study.

## Data Availability

The data that support the findings of this study are available from the corresponding authors upon reasonable request.

## Abbreviations

T3SS: Type III secretion system
Nluc: Nano luciferase
Cya: Calmodulin-dependent adenylate cyclase
NlucN: N-terminal fragment of Nluc
NlucC: C-terminal fragment of Nluc
HiBiT: A NlucC variant
*Pst*: *Pseudomonas syringae* pv. *tomato*
